# Cerebrospinal Fluid Levels of Amyloid Beta 1-43 Mirror 1-42 in Relation to Imaging Biomarkers of Alzheimer’s Disease

**DOI:** 10.3389/fnagi.2017.00009

**Published:** 2017-02-07

**Authors:** Ina S. Almdahl, Camilla Lauridsen, Per Selnes, Lisa F. Kalheim, Christopher Coello, Beata Gajdzik, Ina Møller, Marianne Wettergreen, Ramune Grambaite, Atle Bjørnerud, Geir Bråthen, Sigrid B. Sando, Linda R. White, Tormod Fladby

**Affiliations:** ^1^Division of Medicine and Laboratory Sciences, Institute of Clinical Medicine, Faculty of Medicine, University of OsloOslo, Norway; ^2^Department of Neurology, Akershus University HospitalLørenskog, Norway; ^3^Department of Neuroscience, Faculty of Medicine, Norwegian University of Science and TechnologyTrondheim, Norway; ^4^Preclinical PET/CT, Institute of Basic Medical Sciences, University of OsloOslo, Norway; ^5^AlerisOslo, Norway; ^6^Department of Neurology and Clinical Neurophysiology, University Hospital of TrondheimTrondheim, Norway; ^7^Department of Clinical Molecular Biology (EpiGen), Institute of Clinical Medicine, University of Oslo – Akershus University HospitalLørenskog, Norway; ^8^The Intervention Centre, Oslo University HospitalOslo, Norway

**Keywords:** Alzheimer’s disease, amyloid beta 1-43, cerebrospinal fluid, positron emission tomography, magnetic resonance imaging, mild cognitive impairment

## Abstract

**Introduction:** Amyloid beta 1-43 (Aβ43), with its additional C-terminal threonine residue, is hypothesized to play a role in early Alzheimer’s disease pathology possibly different from that of amyloid beta 1-42 (Aβ42). Cerebrospinal fluid (CSF) Aβ43 has been suggested as a potential novel biomarker for predicting conversion from mild cognitive impairment (MCI) to dementia in Alzheimer’s disease. However, the relationship between CSF Aβ43 and established imaging biomarkers of Alzheimer’s disease has never been assessed.

**Materials and Methods:** In this observational study, CSF Aβ43 was measured with ELISA in 89 subjects; 34 with subjective cognitive decline (SCD), 51 with MCI, and four with resolution of previous cognitive complaints. All subjects underwent structural MRI; 40 subjects on a 3T and 50 on a 1.5T scanner. Forty subjects, including 24 with SCD and 12 with MCI, underwent ^18^F-Flutemetamol PET. Seventy-eight subjects were assessed with ^18^F-fluorodeoxyglucose PET (21 SCD/7 MCI and 11 SCD/39 MCI on two different scanners). Ten subjects with SCD and 39 with MCI also underwent diffusion tensor imaging.

**Results:** Cerebrospinal fluid Aβ43 was both alone and together with p-tau a significant predictor of the distinction between SCD and MCI. There was a marked difference in CSF Aβ43 between subjects with ^18^F-Flutemetamol PET scans visually interpreted as negative (37 pg/ml, *n* = 27) and positive (15 pg/ml, *n* = 9), p < 0.001. Both CSF Aβ43 and Aβ42 were negatively correlated with standardized uptake value ratios for all analyzed regions; CSF Aβ43 average *rho* -0.73, Aβ42 -0.74. Both CSF Aβ peptides correlated significantly with hippocampal volume, inferior parietal and frontal cortical thickness and axial diffusivity in the corticospinal tract. There was a trend toward CSF Aβ42 being better correlated with cortical glucose metabolism. None of the studied correlations between CSF Aβ43/42 and imaging biomarkers were significantly different for the two Aβ peptides when controlling for multiple testing.

**Conclusion:** Cerebrospinal fluid Aβ43 appears to be strongly correlated with cerebral amyloid deposits in the same way as Aβ42, even in non-demented patients with only subjective cognitive complaints. Regarding imaging biomarkers, there is no evidence from the present study that CSF Aβ43 performs better than the classical CSF biomarker Aβ42 for distinguishing SCD and MCI.

## Introduction

Alzheimer’s disease (AD) is the leading cause of dementia. Treatment of this devastating disease will depend on biomarkers that can reliably identify individuals who will develop dementia due to AD in the future. A previous study following patients with mild cognitive impairment (MCI) for 2 years, found that the baseline cerebrospinal fluid (CSF) levels of amyloid-beta 1-43 (Aβ43) could distinguish patients that converted to AD dementia from those that did not, suggesting that CSF Aβ43 could be a useful addition to the more well-studied CSF biomarkers amyloid beta 1-42 (Aβ42), total tau (t-tau), and tau phosphorylated on position 181 (p-tau) (Kandimalla et al., 2011, 2013; Lauridsen et al., 2016). Aβ43 differs from Aβ42 by one C-terminal threonine residue, and is the product of an alternative γ-secretase cleavage pathway from the amyloid precursor protein (APP) (Takami et al., 2009). Findings from studies of neuropathology, genetics and animal models have resulted in the hypothesis that Aβ43 could play a role in AD pathogenesis out of proportion to its low levels in the brain. With its additional C-terminal beta-branched amino acid, Aβ43 could theoretically be expected to be more prone to aggregation than Aβ42. Experiments *in vitro* have yielded conflicting results: some report that Aβ43 indeed aggregates faster than Aβ42 and with a higher potential for seeding aggregation of other Aβ species (Saito et al., 2011; Conicella and Fawzi, 2014), others that Aβ43 aggregates slower with later amyloid nucleation and that it is inefficient in cross-seeding Aβ42 (Chemuru et al., 2016). Whether these experiments reflect the true aggregational process in the human brain is uncertain (Vandersteen et al., 2012). Cerebral deposition of Aβ43 is frequently present both in sporadic and familial AD (Welander et al., 2009; Keller et al., 2010; Sandebring et al., 2013) as a component of both neuritic and diffuse extracellular plaques (Iizuka et al., 1995; Parvathy et al., 2001; Miravalle et al., 2005). Some *PSEN1* mutations associated with familial AD are known to cause an overproduction of Aβ43 (Nakaya et al., 2005; Shimojo et al., 2008). In a transgenic mouse model based on such a *PSEN1* mutation, Aβ43 appeared to have greater neurotoxicity than Aβ42 with short-term memory impairment occurring with rising levels of Aβ43 even before plaque formation (Saito et al., 2011). Aβ43 has also been shown to deposit ahead of Aβ42 in the brain of mutant APP transgenic mice (Zou et al., 2013).

Information is sparse regarding CSF Aβ43 as a potential clinical biomarker, especially in early stages of cognitive impairment. Previously, it has been shown that CSF Aβ43 levels are decreased in MCI and AD dementia as compared to controls, with a strong correlation between CSF levels of Aβ43 and Aβ42 (Kakuda et al., 2012; Lauridsen et al., 2016). At the late stage of dementia, CSF Aβ43 and Aβ42 appear to have equal diagnostic accuracy for discriminating AD dementia from non-demented controls (Bruggink et al., 2013). Clinically more important, however, is the ability of biomarkers to single out non-demented patients that are on a trajectory toward AD dementia. A meta-analysis combining the classical CSF biomarkers; Aβ42 with t-tau and/or p-tau, yielded a mean sensitivity of 84% and a mean specificity of 63% for the distinction between stable and progressive MCI (Ferreira et al., 2014). Enhancement of this diagnostic performance would obviously be an advantage. Lauridsen et al. (2016) found that when used in a ratio with t-tau, substituting Aβ42 with Aβ43 gave a slight, but significant improvement of the diagnostic accuracy for this distinction, a finding that warrants further exploration. The use of CSF biomarkers in clinical routine is impeded by the invasiveness of lumbar puncture and by the high between-center variability particularly in the measurement of Aβ42. Imaging biomarkers are often more readily available, provide complimentary information as well as improve the predictive accuracy for dementia conversion when combined with CSF biomarkers (Vemuri et al., 2009). To our knowledge, CSF Aβ43 has not been described in relation to imaging biomarkers.

Positron emission tomography (PET) imaging allows visualization of cerebral Aβ aggregates *in vivo*. Uptake of amyloid-binding PET tracers, like ^18^F-Flutemetamol (^18^F-FLUT), correlates inversely with CSF Aβ42 levels (Fagan et al., 2006; Li et al., 2015), and positively with Aβ plaque burden observed post-mortem (Ikonomovic et al., 2008). It remains to be determined whether the relationship with amyloid PET is the same for CSF Aβ43. In addition to amyloid pathology, development of AD is characterized by neurodegeneration. Neurodegenerative changes in AD identifiable by magnetic resonance imaging (MRI) include gray matter atrophy of the hippocampus and vulnerable cortical regions (Whitwell et al., 2008; Sabuncu et al., 2011), and microstructural white matter changes resulting in increased mean, radial and axial diffusivity and reduced fractional anisotropy on diffusion tensor imaging (DTI) (Selnes et al., 2013; Amlien and Fjell, 2014; Lee et al., 2015). Several studies have reported correlations between CSF Aβ42 and structural MRI, while others have found no association, with methodological differences suggested as a possible reason for the discrepancy (Vemuri and Jack, 2010; Li et al., 2014). Neurodegeneration is also related to changes in cerebral metabolism as assessed by ^18^F-fluorodeoxyglucose (^18^F-FDG) PET imaging (Fouquet et al., 2009). In AD dementia cortical ^18^F-FDG uptake has been reported to correlate with CSF Aβ42 levels (Vukovich et al., 2009; Yakushev et al., 2012) and ^18^F-FDG PET imaging appears to have high prognostic value in MCI (Shaffer et al., 2013; Perani et al., 2016).

The objectives of this study were to explore firstly whether CSF Aβ43 reflects cerebral amyloid deposits as visualized by ^18^F-FLUT PET, and secondly whether CSF Aβ43 correlates with MRI and ^18^F-FDG PET imaging findings of neurodegeneration in non-demented patients with cognitive complaints.

## Materials and Methods

### Subject Recruitment

#### Cohort 1 – Amyloid PET cohort:

Forty subjects were included in the Dementia Disease Initiation (DDI) project at Akershus University Hospital between March 2013 and March 2016. They were referred by their general practitioner to the hospital’s memory clinic or recruited through newspaper advertisements. Inclusion criteria were complaints of decline in cognitive capacity compared with a previously normal state, age 40–79 and Scandinavian first language. Exclusion criteria were established dementia, neurodevelopmental disorders, known brain injury including recognized previous stroke, as well as any serious somatic or psychiatric disorder or drug use that could significantly influence cognitive capacity. All subjects were assessed with ^18^F-FLUT PET either at the time of inclusion (*n* = 22) or at a second assessment 2 years after first inclusion in the project (*n* = 18). Clinical assessment, lumbar puncture, and MRI were done within 3.5 months of ^18^F-FLUT PET. Thirty-one of the 40 subjects also underwent ^18^F-FDG PET. Patients were interviewed and examined by a physician trained in diagnosing cognitive disorders. A clinical report form was used to collect information about current cognitive symptoms both from the participant and a knowledgeable informant. Standardized cognitive testing, physical examination, and blood screening were completed. MCI (*n* = 12) was defined based on the core criteria in the recommendation from the National Institute on Aging-Alzheimer’s Association (NIA/AA; Albert et al., 2011). Documented impairment greater than expected for the person’s age, gender, and educational level in one or more cognitive domains was operationalized by a score 1.5 standard deviations or more below the normative mean on at least one of the following tests; the delayed recall task of the CERAD Word List Test (Fillenbaum et al., 2008), Trail Making Test B (TMTB) (Reitan and Wolfson, 1985), Controlled Oral Word Association Test (COWAT) (Benton and Hamsher, 1989) and the silhouettes task from the Visual Object and Space Perception (VOSP) Battery (Warrington and James, 1991) or a score below 28 on MMSE (Folstein et al., 1975). All subjects maintained independent functioning in social, and if appropriate, occupational settings, and had a global Clinical Dementia Rating score of ≤0.5 (Morris, 1997). Subjective cognitive decline (SCD) (*n* = 24) was defined according to the recommendations by the Subjective Cognitive Decline Initiative Working Group (Jessen et al., 2014), with normal performance on standardized cognitive tests operationalized by a score above 1.5 standard deviations below the normative mean on the above mentioned tests. Four subjects had been classified as having SCD at inclusion, but did not have cognitive complaints 2 years later when they underwent ^18^F-FLUT PET. They had normal performance on cognitive tests and were classified as cognitively normal with resolution of previous cognitive complaints (CN).

#### Cohort 2 – MRI and DTI cohort:

Fifty subjects were included in the MCI project at Akershus University Hospital between January 2007 and February 2013 after having been referred to the hospital’s memory clinic by their general practitioner. Inclusion criteria were cognitive complaints for at least 6 months and age 40–79. Exclusion criteria included established dementia, major psychiatric disorder, drug abuse, significant solvent exposure, and anoxic brain damage. All subjects underwent lumbar puncture and MRI at inclusion. The subjects were assessed with clinical interview, routine physical examination, blood screening, and a battery of cognitive tests. One subject was found to have been included in Cohort 1 and was therefore excluded from Cohort 2 when data from both cohorts were analyzed together (total number of unique subjects in the study *n* = 89). Subjects in Cohort 2 were defined as having MCI (*n* = 39) if objective cognitive impairment was evident on at least one of the following screening tests; MMSE score below 28, score equivalent to mild impairment on one or more of the items of the Cognistat (Kiernan et al., 1987) or score >1 on I-Flex (Royall et al., 1992). Subjects without objective cognitive impairment on the same screening battery were classified as having SCD (*n* = 11).

### Ethics Statement

The study was conducted in accordance with the Helsinki Declaration. All participants gave written informed consent. The Regional Committee for Medical and Health Research Ethics, South East Norway, approved the study (approval 2009/2550 and 2013/150).

### CSF Collection and Storage

Lumbar puncture was performed generally between 8 a.m. and noon, at the L3/L4, L4/L5, or L5/S1 interspace and without any serious adverse events. The first 4 ml CSF was used for routine clinical investigations. The next 1.5 and 4.5 ml CSF were collected in two polypropylene tubes and centrifuged at 2000 × *g* for 10 min within 4 h of collection. The 1.5 ml CSF was stored at -80°C prior to analysis of the traditional CSF biomarkers Aβ42, t-tau and p-tau. In Cohort 1, the 4.5 ml CSF was aliquoted into 450 μl polypropylene tubes before storage at -80°C, while in Cohort 2 the 4.5 ml CSF was stored at -80°C, before later being thawed and aliquoted, with further storage at -80°C prior to determination of Aβ43. Consequently, the samples underwent one freeze-thaw cycle before determination of Aβ43 in Cohort 1 and two in Cohort 2, with the exception of two samples in Cohort 2 where due to lack of CSF in the biobank, remaining CSF after analysis of the traditional CSF biomarkers was used also for analysis of Aβ43, resulting in three freeze-thaw cycles.

### ELISA Assays and *APOE* Genotyping

Cerebrospinal fluid levels of Aβ42, t-tau, and p-tau were quantified with commercially available ELISAs; Innotest^®^ β-amyloid 1–42 (Vanderstichele et al., 2000), Innotest^®^ hTau Ag (Blennow et al., 1995), and Innotest^®^ phosphoTau (181P) (Vanmechelen et al., 2000) (Fujirebio Europe, Gent, Belgium), and carried out in accordance with the manufacturers’ instructions at the national reference laboratory for these tests at the Department of Interdisciplinary Laboratory Medicine and Medical Biochemistry, Akershus University Hospital. The laboratory lists the following cut-off values for abnormality (modified from Sjögren et al., 2001); t-tau > 300 pg/ml for age < 50 years, >450 pg/ml for age 50–69 years, and >500 pg/ml for age ≥ 70 years, p-tau ≥ 80 pg/ml and Aβ42 < 550 pg/ml.

Aβ43 in CSF was analyzed at the laboratory of the Department of Neuroscience, Norwegian University of Science and Technology, Trondheim, Norway, with an ELISA monoplex kit; Aβ1-43, RE59711 (IBL, Hamburg, Germany) run according to the instructions given by the manufacturer. The antibodies included in the kit were anti-human Aβ (38–43) rabbit IgG affinity purity and anti-human Aβ (82E1) mouse IgG MoAb Fab’ affinity purity. According to the manufacturers of the kits the cross-reactivity for Aβ42 in the Aβ43 ELISA is <1% and the antibodies in the Innotest^®^ β-amyloid 1–42 have 50x less affinity for Aβ43 compared to Aβ42. Samples of CSF were analyzed undiluted and in duplicate. The measurement range for the kit was reported to be 2.34–150 pg/ml. All samples analyzed in the study (7.51–66.49 pg/ml) fell within this range. Intra- and inter-assay variations have been reported previously (Lauridsen et al., 2016). As this study continued from the previously published material, these values were not calculated again.

Apolipoprotein E (*APOE*) genotyping was performed on EDTA blood samples from all subjects at the Gene Technology Division, Department of Interdisciplinary Laboratory Medicine and Medical Biochemistry, Akershus University Hospital according to the laboratory’s routine protocol using real-time PCR combined with a TaqMan assay (Applied Biosystems, Thermo Fisher Scientific, Waltham, MA, USA).

### MRI Imaging Acquisition and Processing

In Cohort 1, MRI scans were acquired on a Philips Achieva 3 Tesla system. A single 3D turbo field echo sequence was acquired for morphometric analysis with the following sequence parameters: TR/TE/TI/FA = 4.5 ms/2.2 ms/853 ms/8°, matrix = 256 × 213, 170 slices, thickness = 1.2 mm, in-plane resolution of 1 mm × 1.2 mm. In Cohort 2, MRI was performed on a Siemens Espree 1.5 T scanner. One 3D magnetization-prepared rapid gradient echo T1-weighted sequence was obtained with the following specifications: TR/TE/TI/FA = 2400 ms/3.65 ms/1000 ms/8°, matrix = 240 × 192, 160 slices, thickness = 1.2 mm, in-plane resolution of 1 mm × 1.2 mm. The pulse sequence used for DTI was: *b* = 750, 12 directions repeated five times, five b0-values per slice, TR = 6100 ms, TE = 117 ms, number of slices = 30, slice thickness = 3 mm (gap = 1.9 mm), in-plane resolution = 1.2 × 1.2 mm^2^, bandwidth = 840 Hz/pixel. Cortical reconstruction and volumetric segmentation was performed with the FreeSurfer image analysis suite version 5.3.0^1^. This includes segmentation of the subcortical white matter and deep gray matter volumetric structures (Fischl et al., 2002) and parcellation of the cortical surface (Fischl et al., 2004) according to a previously published scheme labeling cortical sulci and gyri (Desikan et al., 2006), and thickness values are calculated over the cortical mantle. The thickness value of the entorhinal cortex (ERC) was calculated using a method based on ultra-high resolution *ex vivo* applied to *in vivo* MRI, as implemented in FreeSurfer (Fischl et al., 2009). In addition to hippocampal volume and cortical thickness of the ERC, the thickness of the following cortical regions of interest (ROIs) known to be atrophic relatively early in the development of AD were selected for analysis; the temporopolar, middle temporal, posterior cingulate, inferior parietal, and inferior frontal cortex. For analyses of the total hippocampal volume, the sum of the right and left hippocampal volumes as permillage (‰) of the estimated total intracranial volume was used. For each cortical ROI, the average of the measurements from the right and left hemisphere was used. Image processing for DTI has been described previously (Kalheim et al., 2016). DTI data were missing for one subject (*n* = 49). The fractional anisotropy, mean, radial, and axial diffusivities were assessed in the following four tracts, selected based on previous reports of DTI changes in AD and MCI (Amlien and Fjell, 2014; Lee et al., 2015) and calculated as an average of the metrics from the right and left hemispheres; the cingulum bundles (average of the cingulum-cingulate gyrus bundle and the cingulum-angular bundle), the corpus callosum-forceps bundles (average of the corpus callosum bundles to forceps major and minor, respectively), the uncinate fasciculus and the corticospinal tract.

### PET Imaging Acquisition, Processing, and Interpretation

In Cohort 1 ^18^F-FLUT and ^18^F-FDG PET/CT imaging were performed on the same GE Discovery 690 PET/CT scanner on two separate days. Subjects received a bolus injection of 185 MBq (5 mCi) tracer and after resting were positioned head-first supine in the scanner. A low-dose CT scan was acquired first for attenuation correction. Subjects fasted at least 6 h in advance and blood glucose was measured routinely before ^18^F-FDG injection (all subjects had blood glucose below 8.0 mmol/l). PET scanning in 3D-mode commenced 45 min after injection of ^18^F-FDG and 90 min after ^18^F-FLUT. PET data were acquired for 10 min for ^18^F-FDG and for 20 min (four frames of 5 min) for ^18^F-FLUT. Acquired data were corrected for random events, dead time, attenuation, scatter, and decay. PET volumes were reconstructed with an iterative algorithm (VUE Point FX SharpIR with six iterations, 24 subsets for ^18^F-FDG, four iterations, 16 subsets for ^18^F-FLUT) and smoothed with a post-reconstruction 3D Gaussian filter of 3 mm full-width at half maximum. Image format for ^18^F-FDG was 256 × 256 (pixel size 1 mm × 1 mm), for ^18^F-FLUT 192 × 192 (pixel size 1.3 mm × 1.3 mm), with slice thickness 3.75 mm. In Cohort 2 ^18^F-FDG PET/CT-scans were acquired as previously described (Coello et al., 2013).

Visual interpretation of the ^18^F-FLUT images was done by trained readers and the scans were classified as positive or negative in line with the manufacturer’s guidelines. For the automated quantitative assessment, motion correction of the dynamic ^18^F-FLUT PET was performed using frame by frame rigid registration, then the frames were summed to a single time-frame image and registered to the anatomical MRI volume using a six-parameter rigid registration as implemented in the Spatial Parametrical Mapping (SPM 12, Wellcome Trust Centre for Neuroimaging, UCL, UK) toolbox. Due to missing dynamic images one subject had to be excluded from the automated quantitative analyses (*n* = 39). Five cortical ROIs known to harbor substantial amyloid plaques in AD were selected for analysis of ^18^F-FLUT uptake: the precuneus and posterior cingulate combined, anterior cingulate, prefrontal, inferior parietal and lateral temporal cortex. The average ^18^F-FLUT uptake in each of these ROIs was calculated incorporating values from both hemispheres. The average uptake in the cerebellar cortex, which is usually devoid of amyloid pathology in early AD, was used as the reference region after eroding voxels at the segmentation boundaries to avoid influence due to inaccurate segmentation or co-registration. Regional standardized uptake value ratios (SUVRs) for ^18^F-FLUT were created by dividing the average uptake in each ROI by the average uptake in the cerebellar cortex.

The same ROIs that were used for the structural MRI analyses were selected for study of ^18^F-FDG activity. Uptake in the cerebellar white matter was used as the reference region after first eroding the cerebellar white matter mask. SUVRs were calculated by dividing the average uptake of ^18^F-FDG per voxel in each ROI to the average uptake in the cerebellar white matter.

### Statistical Analysis

The statistical analyses were performed using IBM SPSS version 23 (Chicago, IL, USA) unless otherwise stated. All tests were two-sided and *p*-values below 0.05 were considered significant. Distribution of the variables and whether normal distribution could be assumed were assessed by histograms and a Shapiro–Wilk test. Levene’s statistics were calculated to assess the homogeneity of variance in each variable for parametric tests. For comparisons of CSF biomarker levels, demographical data and neuropsychological test results between SCD and MCI, a χ^2^ test was used for categorical variables, an independent sample *t*-test for continuous variables with normal distribution, and a Mann–Whitney *U* test for continuous variables with non-normal distribution. Binary logistic regression models were created with the SCD/MCI distinction as the dependent variable and with either CSF tau, p-tau or one of the imaging biomarkers as a covariate. In significant models, CSF Aβ43 and Aβ42 were then in turn added as a second covariate. Bivariate correlation and partial correlation controlling for age were assessed between CSF biomarkers, MRI, ^18^F-FLUT and ^18^F-FDG variables. Spearman’s rank coefficients (*rho*) of the correlations between an imaging variable and Aβ43, and the imaging variable and Aβ42, were compared with an asymptotic *z* test using software available from http://quantpsy.org (Lee and Preacher, 2013). To detect potential interrelating effects of aging, all analyses were done both unadjusted and with age as a covariate. Controlling for gender and educational length was not done, as these factors were not found to have significant impact in linear regression models of imaging measures as a function of Aβ. *APOE* genotype was related to CSF Aβ43 levels, but was not found to be a significant factor in regression models that already included either Aβ42 or Aβ43, and was therefore not included as a covariate. Differences in baseline CSF Aβ43 and Aβ42 levels between groups based on the result of the ^18^F-FLUT PET were assessed using an independent samples *t*-test. Receiver operating characteristic (ROC) curves for the prediction of a positive ^18^F-FLUT scan were plotted for both Aβ peptides, and area under the curve (AUC) was calculated. Cut-off values for Aβ43 and Aβ42 yielding the best combination of sensitivity and specificity, were determined by maximal Youden’s index. Differences in AUC were assessed using MedCalc statistical software (MedCalc software, Mariakerke, Belgium).

## Results

### Demographics, Cognition, and CSF Biomarkers: Comparison between SCD and MCI

**FIGURE 1:**
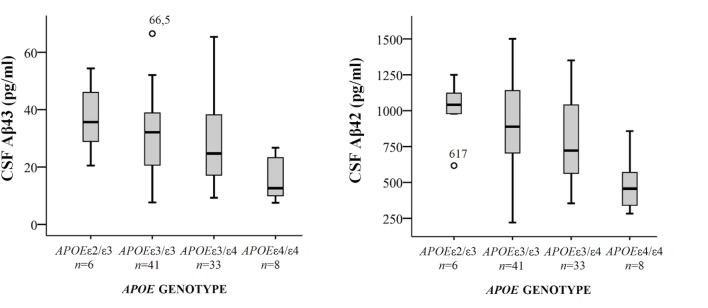
**Box-plots of cerebrospinal fluid (CSF) Aβ43 and Aβ42 according to *APOE* genotype.** All subjects from both cohorts have been included, except for one subject with genotype *APOE*ε2/ε4. CSF Aβ43 for this subject was 17 pg/ml and CSF Aβ42 598 pg/ml. The whiskers represent the range, except for the two outliers. There were significant differences in mean CSF Aβ43 and Aβ42 levels between the *APOE*ε4/ε4-group and the three other groups (all *p* < 0.01). *APOE*, Apolipoprotein E genotype.

Demographical characteristics, cognitive scores and CSF biomarker levels in the SCD and MCI groups are reported in **Table 1**. The frequency of the *APOE*ε4 genotype was similar in both SCD and MCI. The pattern of relative levels of the two CSF Aβ peptides was similar with respect to *APOE* genotype, with significantly lower mean peptide levels in the group with *APOE*ε4/ε4 (**Figure 1**). No significant difference for the correlations between *APOE* allele and Aβ43 and Aβ42 levels was found. There was a strong positive correlation between the CSF measurements of Aβ43 and Aβ42 (all subjects *rho* 0.88, *p* < 0.001) with no significant difference between the groups (SCD *rho* 0.81 and MCI *rho* 0.86). In the MCI group both Aβ43 and Aβ42 correlated inversely with t-tau and p-tau without any significant difference between the two amyloid peptides (MCI *n* = 51, Aβ43:t-tau *rho* -0.45, *p* = 0.001, Aβ42:t-tau *rho* -0.35, *p* = 0.01, Aβ43:p-tau *rho* -0.38, *p* = 0.007, Aβ42:p-tau *rho* -0.40, *p* = 0.003). In the SCD group, however, there were no significant correlations between Aβ43/Aβ42 and t-tau and p-tau. For all subjects (*n* = 89) the overall correlation coefficients for Aβ43:t-tau was *rho* -0.28, Aβ42:t-tau *rho* -0.25, Aβ43:p-tau *rho* -0.29, and for Aβ42:p-tau *rho* -0.37, without any significant differences between Aβ43 and Aβ42. Adjustment for age did not significantly change the correlations between the CSF biomarkers. In binary logistic regression models for the distinction between SCD and MCI, both Aβ43, Aβ42, and p-tau were statistically significant predictors when entered into the model as the only CSF biomarker. In a multivariate model with p-tau, inclusion of Aβ43 added significantly to the prediction, while Aβ42 did not (**Table 2**).

**Table 1:** Demographics, cognitive scores, and cerebrospinal fluid (CSF) biomarkers in SCD and MCI.

	SCD		MCI		*p*
*n*	34		51		
Gender m/f, *n*	15/19		22/29		–
Age	64.5	[9]	65	[10]	–
Years of education	14	[4]	14	[5]	–
*APOE*ε4 (%)	47		45		–
MMSE *total score*	29	[1]	28	[2]	<0.001
RAVLT delayed recall *t-score*	57	[20]	47	[18]	<0.001
TMT B *t-score*	49	[9]	44	[11]	0.005
COWAT *t-score*	52	[13]	49	[16]	–
CSF Aβ43 *pg/ml*	37	[22]	24	[19]	0.004
CSF Aβ42 *pg/ml* (% below cut-off)	981	[488] (12)	679	[388] (26)	0.009 (0.17)
CSF t-tau *pg/ml* (% above cut-off)	312	[155] (6)	335	[267] (31)	0.13 (0.006)
CSF p-tau *pg/ml* (% above cut-off)	57	[25] (6)	69	[34] (35)	0.001 (0.003)


*Data are presented as the median [interquartile range], unless otherwise stated. The cut-off values for CSF Aβ42, t-tau, and p-tau are those of the national reference laboratory: Aβ42 < 550 pg/mL, p-tau ≥ 80 pg/mL, and t-tau > 300 pg/mL for age < 50 years, >450 pg/mL for age 50–69 years, and >500 pg/mL for age ≥ 70 years. APOEε4 is presented as the percentage of subjects with at least one ε4-allele. The scores on TMTB, COWAT, and RAVLT were missing for one subject in Cohort 2. “–” p-value > 0.15. RAVLT, Rey Auditory Verbal Learning Test (Schmidt, 1996); TMT B, Trail Making Test B; COWAT, Controlled Oral Word Association Test; MCI, Mild cognitive impairment; SCD, subjective cognitive decline; APOE, Apolipoprotein E genotype.*

**Table 2:** Specification of logistic regression models for the SCD/MCI distinction with one or two CSF biomarkers as covariates together with age.

Model	χ^2^	Nagelkerke R^2^	Sensitivity	Specificity		OR	*p*
Aβ43	10.2, *p* = 0.006	0.15	88%	50%		0.95	0.004
Aβ42	8.6, *p* = 0.01	0.13	88%	44%		0.998	0.008
p-tau	10.8, *p* = 0.005	0.16	80%	50%		1.03	0.008
t-tau	3.4, *p* = 0.18						
p-tau + Aβ43	15.9, *p* = 0.001	0.23	84%	56%	p-tau	1.03	0.03
					Aβ43	0.96	0.03
p-tau + Aβ42	13.8, *p* = 0.003	0.20	88%	53%	p-tau	1.02	0.04
					Aβ42	0.998	0.09


*OR, Odds ratio; MCI coded 1, SCD 0. n = 85.*

### Amyloid PET

Based on visual interpretation nine of the 40 ^18^F-FLUT PET scans were deemed to be positive (five SCD, four MCI), two borderline positive (one CN and one MCI), two borderline negative (one SCD and one MCI), and 27 negative (18 SCD, six MCI, three CN). The mean CSF Aβ43 in these four groups were 15, 22, 32, and 37 pg/ml, respectively, and the mean CSF Aβ42 547, 657, 1074, and 1042 pg/ml. The mean difference [95% CI] in CSF concentration between subjects with positive and negative scans was 22 pg/ml [13,31] for Aβ43 and 495 pg/ml [380,611] for Aβ42, *p* < 0.001 for both. ROC curves for prediction of a positive scan gave AUC 0.97 for both Aβ43 and Aβ42. The best cut-off value was ≤24 pg/ml for Aβ43 with sensitivity 100% and specificity 93% for a positive scan, and ≤679 pg/ml for Aβ42 with sensitivity 100% and specificity 89%. In the group with negative scans, 16/27 (59%) had *APOE* genotype *APOE*ε3/ε3 and 9/27 (33%) *APOE*ε3/ε4, the same in the group with positive scans was 2/9 (22%) and 5/9 (56%), but the differences were not statistically significant. ^18^F-FLUT SUVR based on the automated quantitative assessment of the scans was not a significant predictor of the SCD/MCI distinction in binary logistic regression models with and without age as a covariate. Mean overall ^18^F-FLUT SUVR for the five ROIs was 1.29, 95% CI [1.17–1.40] in the SCD group and 1.40, 95% CI [1.23–1.58] in the MCI group (*p* = 0.26). There were highly significant inverse correlations between CSF Aβ43 and ^18^F-FLUT SUVR in all the examined ROIs and the correlations became stronger with adjustment for the effect of age (**Table 3**). The same was true for Aβ42, and there were no significant differences between the correlation coefficients for the two Aβ peptides. The correlations with overall ^18^F-FLUT SUVR remained strong also when analyzing only subjects diagnosed with SCD (*n* = 23; *rho* -0.64, *p* = 0.001 for both Aβ peptides, adjusted for age *rho* -0.69, *p* < 0.001 for Aβ43, and *rho* -0.67, *p* = 0.001 for Aβ42). When excluding subjects with visually interpreted definitely positive scans from the analysis (*n* = 31), there were still significant correlations with overall SUVR; for Aβ43 unadjusted *rho* -0.37, *p* = 0.04, adjusted for age *rho* -0.52, *p* = 0.004, for Aβ42 unadjusted *rho* -0.39, *p* = 0.03, adjusted for age *rho* -0.51, *p* = 0.004. CSF t-tau and p-tau were not significantly correlated with overall ^18^F-FLUT SUVR (t-tau *rho* 0.29, *p* = 0.07, p-tau *rho* 0.24, *p* = 0.15).

**Table 3:** Correlation analyses between ^18^F-FLUT SUVRs and Aβ43 or Aβ42 in CSF, unadjusted and adjusted for age.

	Unadjusted		Adjusted for age	
		
	Aβ43	Aβ42	Aβ43	Aβ42
Prefrontal SUVR	-0.60^∗∗^	-0.64^∗∗^	-0.71^∗∗^	-0.73^∗∗^
Precuneus – Posterior cingulate SUVR	-0.67^∗∗^	-0.70^∗∗^	-0.74^∗∗^	-0.75^∗∗^
Anterior cingulate SUVR	-0.61^∗∗^	-0.67^∗∗^	-0.71^∗∗^	-0.75^∗∗^
Inferior parietal SUVR	-0.65^∗∗^	-0.70^∗∗^	-0.72^∗∗^	-0.76^∗∗^
Lateral temporal SUVR	-0.54^∗^	-0.59^∗∗^	-0.64^∗∗^	-0.67^∗∗^
Average of the five SUVRs	-0.63^∗∗^	-0.67^∗∗^	-0.73^∗∗^	-0.74^∗∗^


*Data presented are Spearman’s rank coefficients for correlations between ^18^F-FLUT SUVRs (standardized uptake value ratios) and CSF levels of Aβ43 and Aβ42, respectively. Dynamic ^18^F-FLUT data were missing for one subject, *n* = 39. ^∗^Correlations with *p*-values < 0.001. ^∗∗^Correlations with *p*-values < 0.0001. There were no statistically significant differences in correlation coefficients between CSF Aβ43 and Aβ42.*

### Hippocampal Volume and Cortical Thickness

Magnetic resonance imaging data from the two cohorts were analyzed separately due to the use of different scanners for the imaging acquisition. There were no significant differences in mean hippocampal volume or thickness in the six cortical ROIs between SCD and MCI in either of the cohorts (Supplementary Table) and none of the ROIs were significant predictors of the SCD/MCI distinction in binary logistic regression models even after adjustment for age. In Cohort 1 there were no significant correlations between Aβ43/42 CSF levels and the structural MRI measurements. In Cohort 2 there were unadjusted moderate positive correlations for both CSF Aβ43 and Aβ42 with total hippocampal volume, thickness of the middle temporal, inferior parietal and inferior frontal cortices with statistical significance after correction for multiple testing in seven ROIs. CSF Aβ43 correlated significantly also with thickness of the ERC and CSF Aβ42 with posterior cingulate cortical thickness. After adjustment for effects of age, however, only the correlations with hippocampal volume, inferior parietal and inferior frontal cortical thickness were nominally significant (**Table 4**). In the SCD group CSF Aβ43 correlated significantly with hippocampal volume and CSF Aβ42 with thickness of the posterior cingulate cortex. In the MCI group there were no significant correlations after correction for age. None of the correlations were significantly different between CSF Aβ43 and Aβ42, there was only a trend toward the correlation coefficient for hippocampal volume being stronger with CSF Aβ43 than Aβ42 (*p* = 0.05 unadjusted for age, *p* = 0.07 adjusted for age) in the SCD group. Both CSF Aβ43 and Aβ42 were significant predictors of hippocampal volume in linear regression both with and without age in the model. T-tau was a significant predictor when modeled alone and with age, but not when either of the CSF Aβ peptides were entered into the same model.

**Table 4:** Correlations between CSF Aβ43, CSF Aβ42, hippocampal volume, and cortical thickness in SCD and MCI subjects together or separately in Cohort 2.

		All		SCD *n* = 11		MCI *n* = 39	
				
		Aβ43	Aβ42	Aβ43	Aβ42	Aβ43	Aβ42
Hippocampus volume, ‰		0.52**	0.52**	0.66^∗^	0.26	0.51^∗∗^	0.51^∗∗^
	Age-adjusted	0.33*	0.30*	0.64^∗^	0.29	0.28	0.25
Entorhinal cortex thickness		0.39**	0.29*	0.29	-0.05	0.35^∗^	0.23
	Age-adjusted	0.25	0.10	0.20	-0.09	0.23	0.07
Posterior cingulate cortex thickness		0.37*	0.39**	0.47	0.66^∗^	0.23	0.28
	Age-adjusted	0.22	0.23	0.43	0.72^∗^	0.02	0.04
Temporopolar cortex thickness		0.20	0.27	0.29	0.17	0.13	0.24
	Age-adjusted	0.05	0.12	0.24	0.17	-0.07	0.04
Middle temporal cortex thickness		0.46**	0.51**	0.52	0.34	0.43^∗∗^	0.51^∗∗^
	Age-adjusted	0.27	0.32*	0.49	0.39	0.18	0.26
Inferior parietal cortex thickness		0.50**	0.52**	0.55	0.45	0.47^∗∗^	0.51^∗∗^
	Age-adjusted	0.30*	0.29*	0.54	0.56	0.22	0.24
Inferior frontal cortex thickness		0.41**	0.41**	0.43	0.34	0.39^∗^	0.40^∗^
	Age-adjusted	0.32*	0.31*	0.42	0.34	0.25	0.24


*Data presented are Spearman’s rank coefficients for bivariate correlation and partial correlation correcting for age. *n* = 50. ^∗^Nominal significance (*p* < 0.05), ^∗∗^Significant correlations with correction for multiple testing in seven regions of interest (*p* < 0.05/7). None of the correlation coefficients were significantly different between CSF Aβ43 and Aβ42.*

### Diffusor Tensor Imaging

Diffusor tensor imaging was only available for Cohort 2. There were no statistically significant differences in the DTI metrics of the selected tracts between the SCD and MCI groups and in logistic regression models none of the DTI metrics were significant covariates for the SCD/MCI distinction even after adjustment for age. Both CSF Aβ43 and Aβ42 were inversely correlated with axial diffusivity in the corticospinal tract and this was the only correlation that maintained significance after correction for multiple testing. Both Aβ peptides showed a nominal significant negative correlation with mean diffusivity in the cingulum bundles and corticospinal tract. There was also a nominal significant negative correlation for CSF Aβ42 and radial diffusivity, but a positive correlation with fractional anisotropy in the cingulum bundles. Comparing the two Aβ peptides, the positive correlation with fractional anisotropy in the cingulum bundles was slightly stronger for CSF Aβ42 compared to CSF Aβ43, though it did not reach significance after strict Bonferroni correction for multiple testing (**Table 5**).

**Table 5:** Correlations between CSF Aβ43, CSF Aβ42, and diffusion tensor imaging metrics in the selected tracts.

	Unadjusted			Adjusted for age		
		
	Aβ43	Aβ42	*p*	Aβ43	Aβ42	*p*
FA Cingulum	0.35*	0.53**	0.007^∗^	0.18	0.40*	0.01^∗^
FA Corticospinal	0.11	0.17	–	-0.10	-0.06	–
FA Callosum-Forceps	0.21	0.30*	–	-0.02	0.06	–
FA Uncinate fasciculus	-0.02	0.01	–	-0.19	-0.18	–
DR Cingulum	-0.43**	-0.55**	–	-0.25	-0.39*	–
DR Corticospinal	-0.29*	-0.32*	–	-0.11	-0.12	–
DR Callosum-Forceps	-0.29*	-0.33*	–	-0.07	-0.10	–
DR Uncinate fasciculus	-0.04	-0.03	–	0.18	0.23	–
DA Cingulum	-0.40*	-0.32*	–	-0.27	0.16	–
DA Corticospinal	-0.36*	-0.34*	–	-0.43**	-0.42**	–
DA Callosum-Forceps	-0.36*	-0.34*	–	-0.24	-0.21	–
DA Uncinate fasciculus	-0.28	-0.23	–	-0.09	0.004	–
MD Cingulum	-0.45**	-0.52**	–	-0.30*	-0.37*	–
MD Corticospinal	-0.44**	-0.41**	–	-0.34*	-0.30*	–
MD Callosum-Forceps	-0.31*	-0.34*	–	-0.11	-0.12	–
MD Uncinate fasciculus	-0.13	-0.10	–	0.10	0.17	–


*Data presented are Spearman’s rank coefficients for bivariate correlation and partial correlation correcting for age, and significant *p*-values for the test of difference between the correlation coefficients for CSF Aβ43 and Aβ42. Diffusion tensor imaging measurements were missing for one subject, *n* = 49. FA, fractional anisotropy; DR, radial diffusivity; DA; axial diffusivity, MD; mean diffusivity. ^∗^Nominal significance (*p* < 0.05), ^∗∗^Significance with Bonferroni correction for multiple testing (*p* < 0.05/16).*

### Cortical Glucose Metabolism

As the ^18^F-FDG PET scans were obtained on different scanners in the two cohorts, the data were analyzed in each cohort separately. There were no significant differences in overall ^18^F-FDG SUVR between the SCD and MCI groups and adjustment for age did not change this. There were no significant correlations between ^18^F-FDG SUVRs and either CSF Aβ43 or CSF Aβ42 in Cohort 1 (*n* = 28). In the larger Cohort 2 (*n* = 50) both CSF Aβ43 and Aβ42 appeared to be correlated with glucose metabolism in the hippocampus and several of the cortical ROIs, but this changed after correction for the effect of age when only the correlation between CSF Aβ42 and ^18^F-FDG uptake in the entorhinal cortex was significant after correction for multiple testing. Looking at the SCD and MCI subjects separately, the correlation between CSF Aβ43 and ^18^F-FDG uptake in the posterior cingulate cortex was nominally significant after correction for age, while the only significant correlation after correction for multiple testing was that between CSF Aβ42 and ^18^F-FDG uptake in the posterior cingulate cortex in the MCI group (**Table 6**). By direct comparison none of the differences in correlation coefficients between CSF Aβ42 and Aβ43 were statistically significant. Unadjusted there was an inverse relation between p-tau and overall ^18^F-FDG uptake (average of the seven ROIs), but after correction for age there were no significant correlations with either p-tau or t-tau.

**Table 6:** Correlations between CSF Aβ43, CSF Aβ42, and ^18^F-FDG SUVRs in Cohort 2.

		All		SCD *n* = 11		MCI *n* = 39	
				
		Aβ43	Aβ42	Aβ43	Aβ42	Aβ43	Aβ42
Hippocampus		0.32*	0.42**	0.37	0.53	0.35*	0.43**
	Age-adjusted	0.12	0.22	0.39	0.54	0.01	0.08
Entorhinal		0.35*	0.47**	0.26	0.60	0.34*	0.43**
	Age-adjusted	0.25	0.38**	0.29	0.60	0.14	0.24
Posterior cingulate		0.40**	0.53**	0.08	0.30	0.61**	0.69**
	Age-adjusted	0.16	0.31*	0.05	0.30	0.37*	0.46**
Temporopolar		0.28	0.36*	0.22	0.48	0.22	0.31
	Age-adjusted	0.14	0.23	0.26	0.49	-0.03	0.05
Middle temporal		0.37*	0.44**	0.13	0.31	0.40*	0.46**
	Age-adjusted	0.16	0.24	0.16	0.31	0.10	0.14
Inferior parietal		0.42**	0.50**	0.27	0.42	0.51**	0.56**
	Age-adjusted	0.19	0.27	0.30	0.42	0.22	0.24
Inferior frontal		0.25	0.37*	0.06	-0.01	0.38*	0.50**
	Age-adjusted	-0.05	0.09	0.06	-0.01	-0.02	0.13
Average of all six cortical ROIs		0.41**	0.51**	0.07	0.35	0.48**	0.55**
	Age-adjusted	0.21	0.31*	0.09	0.35	0.19	0.26


*Data presented are Spearman’s rank correlation coefficients for bivariate correlations and partial correlations controlling for age. ^∗^Nominal significance (*p* < 0.05), ^∗∗^Significance with Bonferroni correction for multiple testing (*p* < 0.05/7). None of the differences in correlations coefficients between CSF Aβ43 and Aβ42 were statistically significant.*

## Discussion

The interest in CSF Aβ43 as a biomarker first arose from experimental data suggesting that this peptide could be more prone to aggregation than Aβ42, and thus potentially have importance for amyloidogenesis in AD (Saito et al., 2011; Zou et al., 2013; Conicella and Fawzi, 2014; Burnouf et al., 2015). Our results show that CSF Aβ43 levels are inversely correlated with cortical amyloid deposits, even at the stage of SCD and before extensive amyloid pathology is evident. However, results revealed nothing to support the hypothesis that the amyloidogenic impact of Aβ43 is different to that of Aβ42. The strength of the correlation between CSF Aβ42 and amyloid load was comparable in the present study to that reported in other studies (Jagust et al., 2009; Tolboom et al., 2009; Landau et al., 2013; Palmquist et al., 2014). Investigating the potential role of CSF Aβ43 in very early AD pathology is difficult. It has been suggested that CSF Aβ42 levels start to drop prior to the increase in amyloid tracer uptake (Fagan et al., 2006; Mattsson et al., 2015), but contradictory results have also been presented (Landau et al., 2013). ^18^F-FLUT only binds Aβ when it has formed extensive β-sheet formations in insoluble fibrils, and does not bind to the soluble Aβ oligomers that are suggested more likely to be the main neurotoxic culprit in AD (Selkoe and Hardy, 2016). Whether Aβ43 in CSF may have an impact on the quantity and toxicity of oligomers cannot be answered by the current imaging techniques. Recently, the first successful use of a monoclonal antibody-based PET ligand, capable of binding soluble Aβ protofibrils, was demonstrated in two AD mouse models (Sehlin et al., 2016). Future use of similar radioligands in humans could possibly elucidate the impact on oligomers.

Measurements of Aβ43 in CSF have not previously been described in relation to cerebral imaging findings, while CSF Aβ42 has been extensively studied. CSF Aβ42 has been shown previously to correlate with hippocampal volume in several cross-sectional studies (Apostolova et al., 2010; Wang et al., 2015). Some longitudinal studies have reported no association between CSF Aβ42 and hippocampal volume at baseline, but an association with subsequent hippocampal atrophy (Schuff et al., 2009; Tosun et al., 2010; Stricker et al., 2012; Mattsson et al., 2014). Other studies have shown no association either at baseline (de Souza et al., 2012) or longitudinally (Henneman et al., 2009; Tarawneh et al., 2015). One explanation for the inconsistency is that the rates of alteration of analytes in CSF and imaging biomarkers are neither parallel nor linear. As a result the correlations between biomarkers will change over time with disease progression (Insel et al., 2016). Divergences in how the various disease stages are defined will further contribute to this variability. We found that the correlation with hippocampal volume tended to be stronger in the SCD group in Cohort 2, especially for CSF Aβ43. Previous studies have shown that brain amyloid load is related to hippocampal volume in cognitively healthy elderly (Dickerson et al., 2009) and in SCD, but not in MCI and AD (Bourgeat et al., 2010; Chételat et al., 2010a). Similarly, Fagan et al. (2009) found that CSF Aβ42 correlated with whole-brain volume in elderly subjects without cognitive impairment, but not in MCI and AD, suggesting that the association between atrophy and amyloid could be present only early in the disease process. Many studies have described a strong correlation between CSF tau and hippocampal atrophy in MCI and AD (Henneman et al., 2009; Apostolova et al., 2010; de Souza et al., 2012; Tarawneh et al., 2015). In the current study, we found that hippocampal volume was better predicted by either CSF Aβ43 or Aβ42 than by t-tau or p-tau, which is in line with former studies in SCD and healthy elderly individuals. That the result differs from past reports in patients with MCI may possibly be attributed to the younger age of our MCI subjects compared to many of the previously published MCI cohorts.

White matter changes are also known to be related to CSF biomarkers (Amlien and Fjell, 2014). CSF Aβ42 has been shown to be positively associated with fractional anisotropy and inversely with mean diffusivity (Gold et al., 2014; Li et al., 2014) as found also in the current study. Both DTI and ^18^F-FLUT PET have been reported to be superior to the core CSF biomarkers in predicting the conversion from MCI to dementia (Selnes et al., 2013; Shaffer et al., 2013; Perani et al., 2016). Therefore, it was particularly interesting to compare these biomarkers with CSF Aβ43 that in a previous study was suggested to have the same quality. Surprisingly, fractional anisotropy in the cingulum fibers appeared to be better correlated with CSF Aβ42 than Aβ43 and the trend was the same for cortical glucose metabolism.

The current study has several limitations. The use of different MRI and PET-scanners in the two cohorts made direct comparisons between cohorts challenging. Cortical thickness was measured to be higher in Cohort 2 than in Cohort 1 for several of the ROIs even though p-tau levels were on average higher in Cohort 2. Correcting for age resulted in only a slight reduction in the between cohort differences in these ROIs. It is known that differences in scanner field strength and possibly also scanner settings like pulse sequence, can impact on regional cortical thickness measurements (Han et al., 2006; Govindarajan et al., 2014; McCarthy et al., 2015), which may have contributed to the described differences between the cohorts. The cortical thickness measures were obtained by automated segmentation using the freely available and widely used software FreeSurfer. FreeSurfer version, operating system and workstation used in the processing can also impact the cortical thickness measurements (Gronenschild et al., 2012), but were identical for the two cohorts in this study. Some have suggested that there could be a transitional phase in the development of AD with increased thickness of certain cortical areas (Chételat et al., 2010b; Fortea et al., 2011; Molinuevo et al., 2012), but this has mainly been described in pre-clinical stages. Because we suspected a significant scanner effect, the imaging data were analyzed in each cohort separately, with a lower number of subjects in each analysis as a consequence. The cohorts came from somewhat different populations; all subjects in Cohort 2 were consecutively recruited from a memory clinic, while Cohort 1 also included subjects recruited by advertisements. Greater variability due to partly community based recruitment and fewer subjects with abnormal CSF biomarkers could be the reason why no correlation with neurodegenerative imaging biomarkers was found in Cohort 1. The study is also limited by the fact that we only included subjects that had already developed cognitive symptoms (which are only subjective in the case of SCD). We could therefore not assess CSF Aβ43 in pre-clinical stages of AD such as in cognitively normal subjects with positive ^18^F-FLUT PET, nor could we evaluate the impact of biomarkers on the distinction between controls and SCD. This ought to be assessed in future studies.

## Conclusion

In this first description of CSF Aβ43 in relation to imaging biomarkers, we found that CSF levels of Aβ43 are inversely correlated with fibrillary Aβ accumulation in the brain and more weakly positively correlated with biomarkers of neurodegeneration including hippocampal volume. However, none of the studied correlations between CSF Aβ and imaging measurements were significantly different between the two Aβ peptides when controlling for multiple testing. We conclude that in respect to imaging, CSF Aβ43 does not appear to contribute any added value over the well-established CSF biomarker Aβ42 in distinguishing individuals with SCD from those with MCI.

## Author Contributions

IA planned the study, recruited and clinically examined study participants, performed the statistical analyses and wrote the manuscript. CL planned the study and performed ELISA of CSF Aβ43 together with IM. PS and LK processed the MRI and ^18^F-FDG PET images and performed clinical examinations of participants. CC processed the ^18^F-FLUT PET images. BG visually interpreted ^18^F-FDG and ^18^F-FLUT PET scans. MW carried out laboratory work and administered the CSF biobank. RG did neuropsychological assessments of participants. AB supervised imaging acquisition. LW, SS, GB, and TF supervised the project. All authors critically revised and approved the manuscript.

## Conflict of Interest Statement

The authors declare that the research was conducted in the absence of any commercial or financial relationships that could be construed as a potential conflict of interest.

The reviewer IB and handling Editor declared their shared affiliation, and the handling Editor states that the process nevertheless met the standards of a fair and objective review.

## Footnotes

1 http://surfer.nmr.mgh.harvard.edu/
