# Supplementary material for: Cerebrospinal Fluid Levels of Amyloid Beta 1-43 Mirror 1-42 in Relation to Imaging Biomarkers of Alzheimer’s Disease
Source: Front Aging Neurosci. 2017 Feb 7;9:9. doi: 10.3389/fnagi.2017.00009 (PMC5293760; doi:10.3389/fnagi.2017.00009)
Supplement: Supplementary file 1 [file Table_1.pdf]

SUPPLEMENTARY TABLE: Hippocampal volume and cortical thickness values in the SCD and MCI groups in the two cohorts.

	SCD			MCI			SCD vs MCI	
	Cohort 1	Cohort 2	Cohort 1 vs 2 (*p*)	Cohort 1	Cohort 2	*Cohort 1 vs 2 (p)*	*Cohort 1 (p)*	*Cohort 2 (p)*
n	24	10		12	39			
Age	65.5 (9)	63 (15)	-	63.5 (10)	65 (11)	-	-	-
CSF Aβ43	32 (22)	41 (23)	-	24 (21)	24 (18)	-	-	0.01
CSF Aβ42	973 (560)	1002 (482)	-	772 (510)	659 (350)	-	-	-
CSF t-tau	320 (167)	295 (147)	-	329 (207)	335 (293)	-	-	-
CSF p-tau	53 (25)	61 (15)	0.02	53 (23)	74 (31)	0.02	-	-
Hippocampal volume, ‰	5.36 (1.04)	4.85 (0.72)	-	5.38 (1.30)	4.85 (1.16)	-	-	-
Entorhinal cortex, *mm*	3.56 (0.34)	3.46 (0.42)	-	3.59 (0.24)	3.30 (0.46)	0.02	-	-
Posterior cingulate cortex, *mm*	2.17 (0.21)	2.39 (0.30)	0.002	2.17 (0.22)	2.31 (0.26)	0.02	-	-
Temporopolar cortex, *mm*	3.40 (0.42)	3.70 (0.29)	-	3.37 (0.49)	3.65 (0.39)	0.02	-	-
Middle temporal cortex, *mm*	2.50 (0.25)	2.77 (0.31)	<0.001	2.53 (0.36)	2.76 (0.31)	0.003	-	-
Inferior parietal cortex, *mm*	2.08 (0.17)	2.33 (0.19)	<0.001	2.14 (0.18)	2.21 (0.30)	0.03	-	-
Inferior frontal cortex, *mm*	2.19 (0.17)	2.49 (0.29)	<0.001	2.17 (0.18)	2.44 (0.20)	<0.001	-	-

Data presented are median (interquartile range) and significant *p*-values for the comparison between cohorts for the SCD and MCI groups separately and for the comparison between SCD and MCI for each cohort separately. Hippocampal volume is the combined volume of the right and left hippocampus as parts per thousand (‰) of the estimated total intracranial volume. "-" No statistical significance ($p>0.05$).
